# Parental Control and Adolescent Delinquency Based on Parallel Process Latent Growth Curve Modeling

**DOI:** 10.3390/ijerph18178916

**Published:** 2021-08-25

**Authors:** Xiaoqin Zhu, Daniel T. L. Shek

**Affiliations:** Department of Applied Social Sciences, The Hong Kong Polytechnic University, Hong Kong, China; xiaoqin.zhu@polyu.edu.hk

**Keywords:** longitudinal study, late adolescence, Chinese adolescents, parental influence, delinquency

## Abstract

Although ample evidence demonstrates parental influences on delinquent behavior in adolescent years, few studies have examined how change in adolescent delinquency and change in parental behavior are related to each other, particularly in late adolescence. This study utilized survey data collected over three high school years (N = 3074 Grade 10 students; mean age = 15.57, SD = 0.74 at Time 1) to examine how change trajectory of adolescent-reported delinquency is related to change trajectory of adolescent perceived parental behavioral and psychological control using parallel process growth curve modeling. Results revealed that adolescent delinquency level was negatively associated with both parents’ behavioral control and positively associated with parents’ psychological control at Time 1 (Grade 10). However, adolescent delinquency increased in parallel with decreased parental behavioral control, but not a change in psychological control. Initial paternal behavioral control positively predicted a linear increase slope of adolescent delinquency while initial adolescent delinquency level also positively predicted a linear decrease slope of paternal behavioral control. These results highlight the parallel development of parents’ behavioral control and children’s delinquent behavior and delineate the reciprocal influence between paternal behavioral control and adolescent children’s delinquency.

## 1. Introduction

### 1.1. Parental Control

Parental control has been widely recognized as a key parenting dimension and its multifaceted nature has also been increasingly emphasized with reference to two distinct dimensions: behavioral control (BC) and psychological control (PC) [1,2]. Conceptually, BC represents parental practices to monitor and regulate their children’s behavior by setting and enforcing rules, standards, and regulations [2]. Although BC imposes a certain level of restrictions on children’s pursuit of independence, it provides essential parental supervision and guidance for children to comply with and internalize the underlying principles and social norms. With BC, parents know children’s daily routines and whereabouts, thus parents can have timely communication with children if they show unacceptable behavior, which helps to prevent future delinquency. Empirical evidence has shown that parents’ BC is beneficial for child development, and it is negatively associated with multiple developmental problems, such as depression, delinquency, aggression, and addicted behaviors [3,4,5,6,7].

In contrast to BC, PC is an intrusive parenting style that attempts to manipulate children’s thoughts, emotions, and behaviors by inhibiting their autonomy using love withdrawal, guilt induction, personal attack, and invalidation of children’s perspectives [2]. Instead of communicating the appropriateness of children’s behavior with explanations, PC informs children that their thoughts and feelings, and even themselves are unacceptable [8]. This dysfunctional parenting strategy is believed to impede children’s psychological world and needs fulfillment, thus leading to negative developmental outcomes [9]. The relationship between PC and child developmental disadvantages (e.g., low self-concept and emotional functioning, poor well-being, and more problem behaviors) has been documented in different adolescent populations [4,10,11].

### 1.2. Parental Control and Adolescent Delinquency

Among different developmental outcomes, adolescent delinquency has received much attention because of its high prevalence and comorbidity with other developmental issues, such as addictive behavior and depression [12,13,14]. Delinquency among adolescents refers to a variety of misconducts such as fighting, stealing, cheating, and damaging others’ properties, which violate behavioral norms and/or social laws. It has been considered a public issue worldwide as it is closely linked to negative life outcomes in future, such as depression, substance abuse, violence, and unemployment [15,16,17]. Previous research has identified parental behavioral control as a protective factor of delinquency while psychological control as a risk factor [4,6]. In addition to cross-sectional associations, many studies also examined how parental control may predict delinquency over time. For example, Pinquart’s [4] meta-analysis concluded that initial behavioral control predicted decreases in externalizing problems including delinquency while psychological control predicted increases in the problems. Shek and Zhu’s [6] recent study reported similar findings and further revealed that paternal PC significantly predicted the linear growth rate of delinquency in junior secondary school years.

While these longitudinal studies treated adolescent delinquency as a developmental outcome that varies across time, most of them did not take simultaneous parental changes into consideration. Theoretically, parental control also changes over time and parallel development may occur in parental control and children’s delinquency. After entering adolescence, children have a greater need for independence, autonomy, and meaningful social relationships outside the home. On the one hand, adolescents may be more willing to spend time together with peers instead of staying with their parents. This causes difficulties in actively monitoring children’s daily lives and behaviors, resulting in a decline in parents’ behavioral control [18]. On the other hand, parents’ control and children’s growing desire for independence may result in conflicts between them. As such, children become unwilling to self-disclose their daily lives to parents, which also leads to decreased parental control in terms of less parental supervision [19]. Indeed, recent empirical investigations on trajectories of parenting showed a decrease in parental control over time [20,21]. Hence, to have a better understanding of the linkage between parental control and adolescent delinquency, it is necessary to consider trajectories of both constructs.

Furthermore, the directionality of the relationship between parental control and adolescent delinquency remains open. Most existing studies considered parental control a shaping force of the levels and trajectories of adolescent delinquency [6,22]. This practice is in line with the socialization view in that parents are primary socialization agents of children [23]. However, developmental systems theory also emphasizes that adolescents and socialization systems (e.g., parents) have reciprocal impacts on each other [24]. Some scholars advocated that parenting serves as not only socialization action but also reaction [25]. Essentially, Pinquart [4] reported in his meta-analysis review that initial adolescent externalizing behaviors predicted subsequent increase in dysfunctional parenting (i.e., psychological control and harsh parenting) and decrease in positive parenting (e.g., behavioral control and parental warmth). Recently, Zhu and Shek [26] also found that children’s delinquent and risky behaviors led to decreased parental involvement. These findings echo the coercion theory, which suggests a mutual reinforcement between adolescent misbehavior (e.g., delinquency) and dysfunctional parenting (e.g., harsh controlling and insufficient involvement) [27,28].

Indeed, a handful of empirical studies yielded significant reciprocal effects between parental control and adolescent misbehavior [29,30]. Nevertheless, most parenting research “still adheres to a unidirectional perspective in which parents affect youth behavior but are unaffected by it” [31] (p. 1540). Besides, there are studies that identified only child effects but not parental effects [32,33]. Both inadequate research and inconclusive findings call for further investigations on the possible bidirectional relationships between parental control and delinquency among adolescents.

A methodological issue to note is that almost all the extant studies employed “cross-lagged panel model” (CLPM) in testing the reciprocity of parental and child impacts [29,30,32]. CLPM has its advantage in identifying reciprocal predictions across time as it statistically controls confounding effects of each variable’s temporal stability and contemporaneous correlations between variables. However, this analytical method is unable to capture trajectories of variables (e.g., the rate of change) and reveal associations between the trajectories. One approach that can address this limitation is the “parallel-process latent growth curve model” (PP-LGCM). This approach charts trajectories of more than one construct simultaneously, which clarifies whether and how the intercept and growth rate (i.e., slope) of one variable are associated with the parameters (i.e., intercept and slope) of the other. PP-LGCM becomes increasingly popular in recent longitudinal studies on reciprocal effects [34,35].

### 1.3. The Present Study

**Figure 1:**
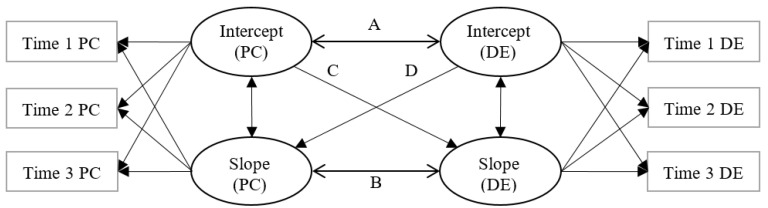
Parallel-process latent growth curve models for parental control (PC) and adolescent delinquency (DE). Error terms and covariates were not shown in the figure. A: cross-sectional associations between baseline PC and baseline DE. B: parallel associations of changes in PC with changes in DE. C: prospective predictions of baseline PC on change rate of DE. D: prospective predictions of baseline DE on change rate of PC.

In response to the needs of addressing the afore-mentioned conceptual and methodological limitations in the extant literature, this study examined the reciprocal causality between trajectories of parental control and adolescent delinquency. Specifically, we conceptualized four PP-LGCMs as shown in Figure 1, with one model for one parent’s behavioral or psychological control. Several paths were estimated simultaneously in each PP-LGCM. First, the intercepts are correlated with each other (i.e., Path A) to indicate a cross-sectional linkage between parental control and adolescent delinquency at baseline. Second, the slope factors are also correlated with each other (i.e., Path B) to show associations between changes (the co-development) in the two constructs. Third, the slope factor of one construct is regressed on the intercept of the other construct (i.e., Path C and Path D) to examine whether the initial level of parental control or adolescent delinquency predicts the rate of change in the other.

In prior studies, parental BC and PC were negatively and positively associated with adolescent delinquency, respectively [4]. Given the positive nature of BC as a parenting strategy, we hypothesized negative associations between parental BC and adolescent delinquency for all paths in the models involving BC.

**Hypothesis** **1a** **(H1a).** *The intercepts of BC and adolescent delinquency would be negatively associated with each other*.

**Hypothesis** **1b** **(H1b).** *The slopes of BC and adolescent delinquency would be negatively associated with each other*.

**Hypothesis** **1c** **(H1c).** *The intercept of BC would negatively predict the slope of adolescent delinquency*.

**Hypothesis** **1d** **(H1d).** *The intercept of adolescent delinquency would negatively predict the slope of BC*.

In contrast, as PC is dysfunctional in nature, we expected positive associations between parental PC and adolescent delinquency for all paths in the models concerning PC.

**Hypothesis** **2a** **(H2a).** *The intercepts of PC and adolescent delinquency would be positively associated with each other*.

**Hypothesis** **2b** **(H2b).** *The slopes of PC and adolescent delinquency would be positively associated with each other*.

**Hypothesis** **2c** **(H2c).** *The intercept of PC would positively predict the slope of adolescent delinquency*.

**Hypothesis** **2d** **(H2d).** *The intercept of adolescent delinquency would positively predict the slope of PC*.

## 2. Materials and Methods

### 2.1. Participants and Procedures

During the 2012/2013 school year (i.e., Time 1), a total of 3973 Grade 10 Chinese adolescents recruited from 28 senior secondary schools in Hong Kong completed a survey investigating adolescent adjustment (e.g., academic performance, internet addiction, delinquent behavior, and well-being) and the psychosocial correlates (e.g., parenting and individual competence such as emotional competence and self-efficacy). The present study only focused on adolescent delinquent behavior and parental control. The students were invited to complete the same survey twice when they were at Grade 11 and Grade 12 (i.e., Time 2 and Time 3), respectively. While the interval between Time 1 and Time 2 is 12 months, it is 10 months between Time 2 and Time 3 as Grade 12 students need to prepare and sit for public examinations in the last several months in their final year of high school.

Ethical review was conducted by the authors’ affiliated institution and ethical approval was obtained. Before questionnaire administration at Time 1, written consent was obtained from the participating schools, students, and their parents. Students completed questionnaires in their classrooms during school time, with the presence of one well-trained research staff in each classroom. The research staff explained the principles upheld in data collection, including confidentiality, anonymity, and free withdrawal, and instructed the participants to respond to all questions based on their own interpretations.

Across the three assessment occasions, completed data were successfully matched among 3074 student participants (Mean age = 15.57, SD = 0.74) at baseline with a composition of 1497 (48.70%) girls and 1577 (51.30%) boys. The majority of these students had intact families (82.2%, n = 2528, 82.2%) and did not live on government welfare (87.3%, n = 2684). This matched sample served as the working sample in the present study.

Attrition analyses were conducted by comparing baselines scores between the working sample (N = 3074) and the dropouts (N = 899). For the baseline social demographic variables at Time 1, no significant differences were observed between the two groups regarding their family intactness and economic condition. However, there was a higher proportion of girls in the matched sample (χ^2^ = 10.26, *p* < 0.01, φ = 0.05). Besides, the matched sample (mean age = 15.57, SD = 0.74) was slightly younger than the dropouts (mean age = 15.88, SD = 0.94, F = 99.05, *p* < 0.001, η^2^_p_ = 0.025). For the parental control and adolescent delinquency at Time 1, adolescents in the two groups reported similar maternal BC (F = 2.50, *p* = 0.11) and paternal PC (F = 1.65, *p* = 0.20). However, students in the working sample rated their fathers’ BC slighter higher (F = 8.98, *p* = 0.003, η^2^_p_ = 0.002) while they rated their mothers’ PC slighter lower (F = 6.27, *p* = 0.012, η^2^_p_ = 0.002) than did the dropouts. Besides, self-reported delinquency level was slightly lower among the matched sample (F = 11.29, *p* < 0.001, η^2^_p_ = 0.004). As the effect sizes of the observed differences in the baseline measures were not large, it can be inferred that attrition did not cause a major bias in our study.

### 2.2. Mesures

#### 2.2.1. Delinquency

Adolescent delinquency was measured using a self-reporting scale, which contained 11 delinquent behaviors that do harm to others or violate regulations or norms, including “stealing,” “cheating others,” “truancy,” “running away from home,” “damaging others’ properties,” “beating others,” “gang fighting,” “speaking foul language,” “staying away from home overnight without parental consent,” “bullying,” and “trespassing.” The participants reported the frequency they showed each of the behaviors in the previous year from “0” (i.e., “never) to “6” (i.e., “more than 10 times”). The average score of the 11 items indicated the participants’ delinquency level. This scale demonstrated acceptable internal consistency and validity (unidimensional factorial validity and construct validity) in past research involving Chinese adolescents [6,26,36,37]. In our study, confirmatory factor analysis (CFA) indicated that the one-factor structure of the scale fit the data adequately across three waves (“comparative-fit index, CFI” = 0.92, “Tucker–Lewis index, TLI” = 0.90, “root mean square error of approximation, RMSEA” = 0.03, “standardized root mean square residual, SRMR” = 0.04, and average factor loading = 0.50 at Time 1; similar results at Time 2 and Time 3). Besides, the Cronbach’s α values of this scale varied between 0.80 and 0.85 across three occasions.

#### 2.2.2. Parental Control

Parental control included BC and PC, which were measured by respective subscales derived from the validated “parent–child subsystem quality scale (PCSQS)” [38]. From “1” (i.e., “strongly disagree”) to “4” (i.e., “strongly agree”), respondents rated both parents’ BC on seven items (sample items: “My father/mother asked me about what I did after school” and “My father/mother actively understands my afterschool activities”) and PC on four items (sample items: “My father/mother often wants to change my mind or feelings for things” and “My father/mother values his/her thoughts and overlooks mine”). The average score of each subscale was computed. These subscales have been widely used to measure Chinese parents’ parenting behaviors and showed adequate psychometric properties [10,39]. In our study, CFA indicated that the two-factor structure (BC and PC) for each parental scale fit the data adequately across waves (Paternal scale at Time 1: CFI = 0.96, TLI = 0.94, RMSEA = 0.08, SRMR = 0.07, and average factor loading = 0.74; Maternal scale at Time 1: CFI = 0.96, TLI = 0.95, RMSEA = 0.08, SRMR = 0.07, and average factor loading = 0.75; similar results at Time 2 and Time 3). The Cronbach’s α estimates of all subscales were above 0.85 across waves.

#### 2.2.3. Covariates

Student age, gender, family intactness and economic condition were covariates in the current study because these variables were associated with parental behaviors and adolescent delinquency in previous studies [6,21,40]. For family intactness, participants whose parents were “in the first marriage” were grouped as “living in intact family” and those students whose parents were “separated,” “divorced,” or “re-married” were considered “living in non-intact family.” For family economic conditions, reliance on “comprehensive social security assistance” (CSSA) provided by Hong Kong Government was used to indicate economic disadvantage.

### 2.3. Data Analysis Procedure

Four PP-LGCMs were conducted using Mplus 8.5 [41], with one model for one parent’s behavioral or psychological control. The general purpose of the analyses is to understand how the trajectory (i.e., intercept and change slope) of one parent’s control (behavioral or psychological control) is related to the trajectory of children’s delinquent behavior over time. Because some participants missed some questions, there were missing values at the variable level. The percentage of missing values in all variables across the three time points ranged between 0 to 4.81%. The “full information maximum likelihood estimation” that utilizes all available information for each participant, was employed to handle the missing at random data in the present study. This method was shown to produce unbiased parameter estimates for LGCM [42,43].

Figure 1 outlines the conceptual diagram of the PP-LGCM models. As shown in Figure 1, a linear latent growth trajectory with a latent intercept and a linear slope were estimated for each parental control factor and adolescent delinquency. For the intercepts, the loadings associated with the means of the respective construct at different assessment points were fixed to 1. Based on the time interval between each two consecutive points, the loadings associated with three assessment occasions were fixed to 0, 1, and 1.83, respectively, for the slope factors of both parental control and delinquency. Four paths (Path A, B, C, and D) were estimated simultaneously in each PP-LGCM, with the effects of covariates statistically controlled by regressing the latent intercepts and slope factors on the four covariates. As findings based on the univariate trajectories of adolescent delinquency and levels of parental control have been separately reported elsewhere [21,40], the present study focused on the multivariate analyses (i.e., associations between the trajectories indicated by the four paths).

Model fit was evaluated by χ^2^, CFI (“comparative fit index”), TLI (“Tucker–Lewis index”), RMSEA (“root mean square error of approximation”), and SRMR (“standardized root mean square residual”). Four criteria were adopted for indicating good model fit, including “CFI ≥ 0.95,” “TLI ≥ 0.95,” “RMSEA ≤ 0.06,” and “SRMR ≤ 0.06” [44].

## 3. Results

Results of the PP-LGCMs are shown in Table 1. With the effects of covariates being statistically controlled, all models fitted the data well. The means of the latent intercepts and linear slopes were statistically significant in all models, indicating that parental control factors and adolescent delinquency changed significantly over time. More specifically, while parental behavioral and psychological control significantly declined over time (mean slopes ranged between −0.031 and −0.018, *p* < 0.01), adolescent delinquency level significantly increased over time (mean slope = 0.022, *p* < 0.001).

As revealed by the significant cross-sectional associations (Path A), higher initial BC of parents was associated with lower initial delinquency among adolescents (father: r = −0.299, *p* < 0.001; mother: r = −0.227, *p* < 0.001) while higher initial PC was linked to higher initial adolescent delinquency (father: r = 0.115, *p* < 0.001; mother: r = 0.151, *p* < 0.001). The findings provided support for H1a and H2a.

Results also revealed negative relationships between the change slopes in parental BC and adolescent delinquency (Path B) (father: r = −0.432, *p* = 0.012; mother: r = −0.230, *p* = 0.04). However, the associations between the change slopes in parents’ PC and children’s delinquency were not significant. Thus, H1b was supported but H2b was not. The results implied that adolescent delinquency increased in parallel with decreased parental behavioral control, but not with change in psychological control.

Finally, for Path C and D, only paternal BC at baseline significantly and positively predicted the change slope of children’s delinquency (β = 0.154, *p* = 0.013) and the intercept of children’s delinquency also significantly and positively predicted the change slope of paternal BC (β = 0.151, *p* = 0.005), indicating reciprocal prediction effects between paternal BC and children’s delinquency over time. However, the direction of effects was just opposite to Hypothesis H1c and H1d. Thus, H1c, H1d, H2c, and H2d were not supported.

## 4. Discussion

Using data collected from Chinese adolescents in Hong Kong across three senior secondary school years, this study investigated the reciprocal relationships between parental control and adolescent delinquency. By applying “parallel-process latent growth curve models” (PP-LGCMs), this study modeled the reciprocal effects as parallel developing processes involving both level and change rate of the constructs. In this regard, the present study adds value to the extant literature by endorsing a bidirectional perspective and extending the research scope from level-level associations based on cross-lagged analyses to associations between trajectories based on multivariate latent growth curve modeling.

Consistent with our hypotheses, negative concurrent associations were observed between both parents’ BC and adolescent delinquency. The findings are in line with the general conclusion that BC benefits child development, which has been widely supported by previous cross-sectional and longitudinal associations between levels of parents’ BC and adolescent problem behavior [4,45,46]. Our investigation expands this relationship pattern to the rate of changes. Specifically, the linear change rates of parents’ BC were negatively related to the change rates in delinquency, suggesting a faster decline rate in parental BC being associated with a steeper increasing slope in adolescent delinquency. The findings support the prediction of the developmental systems theory [24] that parenting strategy (e.g., BC) and adolescent behavior (e.g., delinquency) co-develop across time.

Given the parallel process relationships between BC and adolescent delinquency, it is important to stress the bidirectionality of the effects on each other. In the present study, the initial levels of fathers’ BC and adolescent delinquency positively predicted each other’s change rate over time. The positive pathway was unexpected, indicating that, adolescents with initially high paternal BC had faster increases in delinquency over time compared to those with initial low levels of paternal BC. Likewise, fathers with children who were initially more delinquent showed rapid declines in their BC. The observations maybe related to a floor or ceiling effect that has also been reported in other youth research [47,48]. For example, high initial paternal BC suggests a low initial delinquency level, which would have rooms for increase rapidly. An alternative explanation is the “regression to the mean”, where lower levels of paternal BC and children’s delinquency move up to, while higher levels move down to the mean [49]. The third possibility is that higher paternal control in late adolescence may lead to higher father-adolescent conflict which would eventually lead to higher adolescent delinquency as negative coping.

The unexpected positive reciprocal effects between paternal BC and adolescent delinquency also indicate that, although the initial levels and the linear slopes of the two constructs were negatively associated with each other, the associations decelerated over time. This interpretation is concordant with prior findings that parenting–delinquency association is stronger among younger adolescents [50,51]. As children mature and develop in adolescence, they become increasingly independent and less affected by parents but more attached to alternative social relationships, in particular, peer interactions [52]. For example, some studies found that peer influence appeared to buffer and mediate parental impacts on adolescent development [53,54].

Nevertheless, there were no significant positive reciprocal effects between maternal BC and children’s delinquency, suggesting that changes in maternal BC and child delinquency were not affected by the initial level of the other. Thus, the possible ceiling or floor effect or the “regression to the mean” effect was not that evident among mother-child dyad. Despite the possibility of weakening parental impacts and increasing adolescent autonomy, parent–child interactions, especially mother–child interactions in the present study, still shaped adolescent development to a certain extent [55,56]. This conjecture seems consistent with the general conclusion that mothers are more involved in parenting and they are commonly more controlling [6,10]. Given that the parallel association between adolescent delinquency and maternal BC (i.e., Path B) appeared weaker than that for paternal BC, the latter is more likely to serve as both action and reaction while maternal BC is an active action to a larger extent. As the present findings are novel, future studies are needed to consider the bidirectionality of parent–child interactions by differentiating maternal and paternal impacts.

As expected, positive cross-sectional associations were identified between initial levels of both parents’ PC and adolescent delinquency, which are consistent with the general finding in previous research that PC impedes child development [4,45,46,57]. However, different from BC, parental PC did not change in parallel with the change in adolescent delinquency as there were no significant relationships between slopes. The reciprocal effects between PC and adolescent delinquency were not significant either. The present differences in the findings regarding the two types of parental control are particularly interesting. One possibility is that adolescent delinquency as a form of externalizing problem is more closely related to parents’ BC than to parents’ PC. For instance, parental PC was found to be more directly related to children’s internalizing problem while parental BC was directly associated with children’s externalizing problem [46]. Other studies also observed that parents’ reactions to children’s externalizing problems are more likely to be manifested as BC while reactions to children’s internalizing problems have much to do with PC [26,31]. Future studies are needed to replicate the present findings and to examine the different types of parental control and adolescent externalizing as well as internalizing behavior.

## 5. Limitations

Although the current study is pioneering in examining the parallel process relationships between the two types of parental control and children’s delinquency, four limitations are noted. First, the present study only investigated one externalizing problem. To portray a more holistic picture of the bidirectional influences between parents and children over time, it is necessary to consider other externalizing behaviors and even internalizing problems, such as anxiety and depression. Second, the present data were derived merely from adolescents’ self-perception. There is evidence suggests that the type of informants may affect the magnitude of parenting–adolescent problem associations [50,58]. However, one can argue that adolescents may know themselves better than parents, peers, or teachers, and their perceptions and experiences are what really matter in their development process. This may be one of the reasons that child report is widely adopted in youth studies, especially for those involving multiple occasions of data collection. In this case, future studies can further validate the findings based on child-reporting data by involving different informants, such as adolescent participants, their parents, teachers, or peers, and comparing the findings derived from these data. Moreover, adolescent perception of parental control and delinquency does not directly measure the two constructs in behavioral terms. Future studies will also benefit from adopting direct measure of parenting and delinquency (e.g., observation of parental behavior and school record of student misbehavior). Third, it is possible that the current measure did not capture all controlling behaviors of parents, which might contribute to the floor or ceiling effects in the present study. Thus, future studies can also review alternative observations of parental control to verify the present findings. Finally, additional covariates could be considered, such as family environment, family structure, the social and academic environment, and parenting styles. Nevertheless, because there is limited longitudinal Chinese research on family processes and adolescent developmental outcomes [59,60,61], this study is a constructive response to this limitation in the scientific literature.

## 6. Conclusions

This study employed a parallel process approach to examine the reciprocal relationships between linear trajectories of parental control and adolescent delinquency. Based on data collected over a three-year course from more than 3000 Hong Kong Chinese adolescents, our findings support the hypothesis that parental behavioral control co-develop with adolescent delinquency. Besides, while the parallel process relationship associated with paternal behavioral control decelerated over time, the relationship associated with maternal behavioral control did not. Furthermore, the parallel process relationships were not identified for parental psychological control. These differences highlight the importance of differentiating paternal and maternal impacts and different types of parental control.

## Figures and Tables

**Table 1:** Parameter estimates and model fit for the parallel-process latent growth curve models (PP-LGCMs).

Parameter	Father Behavioral Control		Mother Behavioral Control		Father Psychological Control		Mother Psychological Control	
	Estimate	S.E.	Estimate	S.E.	Estimate	S.E.	Estimate	S.E.
Mean								
PC intercept	2.473 ***	0.012	2.899 ***	0.011	2.184 ***	0.013	2.252 ***	0.013
PC slope	−0.024 ***	0.005	−0.031 ***	0.005	−0.018 **	0.008	−0.018 **	0.008
DE intercept	0.501 ***	0.010	0.501 ***	0.010	0.502 ***	0.01	0.502 ***	0.01
DE slope	0.022 ***	0.006	0.022 ***	0.006	0.022 ***	0.006	0.022 ***	0.006
Growth factor associations								
I_(PC)_ ↔I_(DE)_ (Path A)	−0.299 ***	0.027	−0.227 ***	0.027	0.115 ***	0.033	0.151 ***	0.032
S_(PC)_ ↔S_(DE)_ (Path B)	−0.432 *	0.172	−0.230 *	0.112	0.256	0.151	0.362	0.242
I_(PC)_→S_(DE)_ (Path C)	0.154 *	0.062	0.100	0.059	−0.120	0.067	−0.061	0.061
I_(DE)_→S_(PC)_ (Path D)	0.151 **	0.054	0.048	0.048	−0.026	0.066	−0.160	0.109
Model fit								
χ^2^	17.627		30.754		21.091		19.883	
df	15		15		15		15	
CFI	0.999		0.995		0.998		0.998	
TLI	0.998		0.988		0.994		0.996	
RMSEA	0.008		0.019		0.012		0.011	
SRMR	0.007		0.008		0.010		0.009	

Note: covariates were controlled in all models. PC = parental control factor; DE = adolescent delinquency. Double headed arrows represent correlations and single headed arrows represent regression effects. I_(PC)_ = intercept of parental control factor; I_(DE)_ = intercept of adolescent delinquency; S_(PC)_ = slope of parental control factor; S_(DE)_ = slope of adolescent delinquency. S.E. = standard error; df = degree of freedom. * *p* < 0.05; ** *p* < 0.01; *** *p* < 0.001.

## Data Availability

The data presented in this study are available on request from the corresponding author. The data are not publicly available due to privacy.
